# Progressive Paralysis of the Insane

**Published:** 1868-06-15

**Authors:** A. W. Bosworth

**Affiliations:** Thesis for the Degree of M.D., Presented to the Faculty of the Rush Medical College, Chicago; Paris, France


### CHICAGO MEDICAL JOURNAL.

*Vol. XXV.*—*JUNE* 15, 1868.—*No.* 12.

### PROGRESSIVE PARALYSIS OF THE INSANE.

(PARALYSIE GÉNÉRALE DES ALIÉNÉS OF THE FRENCH.)

BY A. W. BOSWORTH, M.A., PARIS, FRANCE.

Thesis for the Degree of M.D., Presented to the Faculty of the Rush Medical College, Chicago.

(*Continued from page* 340.)

NOTWITHSTANDING this variance of opinion, all are agreed that hereditability is a powerful cause, that it plays an important part in this dreadful disease. M. Bayle admits it as not doubtful in more than half of the cases. M. Calmeil reduces this proportion to one third of the cases. This proportion would probably still be diminished if we only sought among the ancestors for general palsy; but all the neuroses, different palsies, etc., are admitted. These causes may be regarded as a predisposition to general palsy for the descendants, but they do not strictly indicate in what proportion general palsy itself is transmissible.

M. Calmeil says that this class of patients have among their ancestors a large number who have died from chronic, diffuse encephalitis; or encephalitis with deep, circumscribed foci; or from congestive affections of the intra-cranial or cerebro-spinal centres. That it is very probable that the nervous system, from a very early date, even from a vicious pre-organization, was affected.

In another place, the same author says that more than one fourth of the patients affected with general palsy, have among their ancestors those who were affected with melancholia, mania, dementia, hemiplegia, or epilepsy. It is a question worthy of consideration, and which we should be happy to discuss if time and space were here admissible, whether procreation at an advanced age of the parents, and consanguineous marriages, are conditions which augment the frequency of the hereditability in general palsy, as they are usually admitted to do in madness. There is one condition which would tend to diminish the frequency of hereditary transmission of general palsy : *i. e.*, this disease is comparatively seldom seen with woman, whilst it is very common with man. It is well known that authors admit hereditability to be more often transmitted from the mother than from the father. To strengthen this latter idea, we can only here mention that Mr. Baillarger has found in 600 cases of mental disease, 453 of which were hereditary, that 271 were transmitted by the mother, and only 182 by the father.

### Temperament.

Its influence appears to be doubtful; yet plethoric persons, from their greater aptability to congestions, those having thick-set limbs, large cavities, the pilous system black and greatly developed, with the muscular system strongly accentuated, are noticed to be the most frequently affected with general palsy.

### Sex.

As a general rule, one fourth of the persons entering the asylums of the insane are men, and only one tenth are women; yet, after Bayle, we find twenty-four cases among females for 182 among males.

After M. Calmeil, who publishes eighty-two observations, there are nine cases among females and seventy-three among males.

The women who are affected are generally from the lower classes, and especially from the class given to prostitution.

Of all, the most exposed is man, vigorous man, who, confiding in his strength, allows himself to be mastered to a great degree by his passions, thus committing both physical and mental excesses, which he foolishly believes do not interfere with his constitution: yet, alas! too often, when least expecting it, he becomes a victim, and is now to be most basely humiliated. For reasons too evident here to be mentioned, let it be observed that the class furnishing the next greatest number to general palsy, is the courtesan. Least of all exposed, it appears, is noble woman in the easy walks of life, placed by wealth above excessive corporal and mental fatigue, and less given to unbounded excesses of the passions.

### Age.

M. Calmeil says he has "never seen this disease select any one before the age of 22; it is rare from 23 to 26; augments rapidly from 27 to 35; very frequent from 35 to 55; from 55 it continues to decrease in frequency." In old age this malady is not seen, but in its place is found cerebral softening of greater or less extent, accompanied with senile dementia. After many authors, the age of 41 is often a critical period, yet we will only remark that the period of commencement of general palsy is very variable.

### Profession.

There are certain professions which inevitably have a tendency to produce cerebral congestion, and thus predispose to our disease. Among them we may mention, wine merchants, distillers, grocers; tobacco merchants, especially those selling at the same time alcoholic drinks; officers of the army, who eat and drink too much; cooks, who are continually exposed to a high temperature, to various gaseous emanations, and often given to drinking; prostitutes, devoted to every pleasure that pleases their palate or their misguided mind; painters, sculptors, and musicians, whose imagination is continually excited, are predisposed to dementia when leading a regular life; but when, with their intellectual fatigue, they become too devoted to Venus, or to alcoholic drink, they are predisposed to general palsy. Finally, it is considered that all intellectual excess causing frequent fatigue, produces a predisposition. In the great centres of population, where all the causes of general palsy seem to unite to produce this frightful disease, the frequency of which is said by many to be increasing, do we find this affection much more often than in small cities and in the country.

Those professions which are sometimes accompanied by intoxication, as alcoholic, or saturnine, undoubtedly have a direct action in producing this disease, as some authors admit; and it is a great question if palsy produced by saturnian influence should have a separate place in nosology.

The distinction between general palsy as produced by lead, and between the same disease as we have described it, are only symptoms; such as coloration of the skin and nails when in contact with a sulphurous bath, or the arthralgia or colic peculiar to the saturnine influence; there are a few other distinctions which disappear in a short time, and then the disease is to march as has been described. The same reasoning also applies to general palsy produced by alcoholic intoxication, as it, when once fully developed, presents the same symptoms, march, termination, and anatomical lesions.

### Climate and Season.

It was believed for some time that general palsy did not exist in hot countries. Since its symptomatology is better known, and since it has been better separated from mental alienation, numerous cases have been found in those countries where its existence had been denied.

Contrary to the opinion of Esquirol, it is found not to be a rare disease in the south of France, in Italy and Spain, yet in these latter countries it appears to be less common than in

Paris and London. Notwithstanding this malady is said to begin or present its paroxysms in autumn, and to generally terminate by death during the same season, yet it appears from the works of M.M. Baillarger, Aubanal, and Thore, that the frequence of general palsy is the same in all seasons.

### Occasional Causes.

Cerebral congestions: M. Bayle considers them as being constant; many authors admit the same opinion. Their character is to be sudden; most often they are preceded, accompanied or followed by a change of disposition, or of character. M. Parchappe considers these congestions as being rather the effect of the disease than its cause. In many cases we should consider the cerebral congestions only as symptoms, and not as causes more or less distant. If a congestion is soon followed by an inequality of the pupils, difficulty of speech, deviation of the tongue, muscular weakness, change of character, etc., we may diagnosticate a case of general palsy which had its beginning anterior to this cerebral congestion, and easily coincide with M. Calmeil, when he says: "This disease sometimes begins by a violent congestive attack, either apoplective or convulsive."

### Sanguineous Suppressions.

Exanthematous suppressions are at least doubtful as a cause. The suppression of hæmorrhoids, of epistaxis, of the menses, has been given by some. As to the suppression of the menstrual flux, it is proved that among the many affections which may result from it, general palsy must be included. A fact worthy of attention, and which may often foreshow an amelioration or a relapse is, that it has been remarked that the menses, suspended during the attacks, reappear during the intermissions, and even during the remissions. Some paralytical women have, during their menses, lost complete control of the canal and vesical sphincters, and thus, being unable to retain the urine and fæces, they are, as they say in France, *gatenses.*

### Excesses.

They may be divided into two classes: 1st, psychical; 2nd, sensual.

Among the first may be arranged that feverish super-activity which impels man to desire rapidly to obtain honors, fortune; to undertake distant voyages, or causes him to begin immense enterprises, which demand an enormous increase of intellectual labor; excesses which bring in their train profound chagrin, such as deceived ambition, or reverse of fortune.

Mortifications, profound and repeated, such as sorrow resulting from broken love, the death of a parent, etc., whatever may be their cause, are a powerful incentive to mental alienation.

In these cases the apparition of simple madness differs from the explosion of the palsy. When this latter arises as a result of some deep chagrin, usually it begins suddenly, in such a manner that there may seem an exaggeration of real sorrow, which even may be considered as a first symptom of that form of the disease known as the melancholical variety.

An excess of the passions has also been given; anger, becoming violence from futile motives, political preoccupations, which are so often reflected in delirium.

### Sensual Excess.

Here should be noticed sensual habits; too much alcoholic stimulant, aside from its producing delirium tremens, is but too well known to be a very frequent cause of general palsy. When a person has become the victim of chronic alcoholism, there may occur a moment when new symptoms, characteristic of our disease, become apparent; and, although it is not always easy to determine the exact date at which begins the cerebral lesion, yet we now behold it making rapid progress towards a fatal termination.

Some authors cite cases of general palsy resulting from an excessive use of tobacco or opium.

Onanism and venereal excesses are powerful and frequent causes. After Calmeil, cerebral congestion is frequent among women devoted to gallantry, and among that numerous class who, debasing the noble qualities of man, divinely given, lead and terminate a life more brutal that that of the beast of the field, by vile prostitution.

Both males and females, living a life of celibacy, are more exposed to general palsy than are married people. Useless to here discuss this point.

Not desiring to enumerate the various diseases of the viscera, enlargements of the aorta, hypertrophies of the heart, which have been given as causes, we would remark that syphilis would appear to be a cause, especially when in the syphilitic diathesis there are cerebral accidents produced by tumors of the cranium or of the brain. As a general rule, we think the paralytic should be considered as such, and if he has syphilis, put it down as a coincidence, a concomitant, and not as the active cause of his palsy, except in rare cases.

In regard to the pellagra, a disease yet too little known, being so powerful a cause as to produce pellagrous palsy, not differing from general palsy either by its symptoms, its course, or its anatomical lesions, as M. Baillarger maintains, it appears not yet to be demonstrated. Because the pellagra may produce vertigo, melancholy, violent delirium, and often not before ten years, torpor and severe convulsions, is it a sufficient reason to conclude that it is analogous to general palsy? More consistent would it be to consider it as a concomitant. If we are to give as causes of this disease all the maladies of the medical nomenclature, when shall its intricacies be made plain?

### Hematoma Auricularia.

Although these tumors are sometimes seen in other nervous diseases, yet especially do they become manifest in general palsy. Their most probable cause is from the great tendency to cerebral congestion during this disease, which may easily increase the intensity of the aural circulation from the intimate relation of the auricular vessels with those of the head. We can here no more than refer for their description to M. Marcé, Paris, 1862, and especially to M. Foville, of the same city.

### Pathological Anatomy.

Before entering into this part of our subject, we feel it to be just to declare that for the following lines we are greatly indebted to the works of M.M. Marcé and Calmeil, of Paris.

Often in this disease the cranium is very thick, and of abnormal density. If the subject has died from congestion, the skin of the head is greatly injected. Various authors have endeavored to explain the symptoms seen in this disease, by starting from different standpoints. Thus M. Bouillaud, reflecting on the want of co-ordination which is remarked in general palsy, believes that the principal lesion is seated in the cerebellum. He has remarked, in analyzing the work of M. Calmeil on "Diffuse Chronic Encephalitis," that this author's observations only related to the cerebrum, and that the cerebellum had been neglected. M. Parchappe, in his writings, has endeavored to reconcile M. Bouillaud's ideas, and subsequently found the cerebellum often interested, and sometimes so much so, that he (M. Parchappe) considers the lesions of this part of the brain as frequently pathognomonic of general palsy at an advanced period. Nevertheless this latter author has found these lesions less often in the cerebellum than in the cerebrum. Pathological anatomy would seem to explain the trouble of speech at the beginning of this disease. After the works of M. Flourens, who locates intelligence in the cerebral lobes, and after M. Bouillaud, who places speech under the dependence of the anterior lobes, we might endeavor to comprehend these symptoms. Indeed, after M. Parchappe, it is on a level with the frontal horns that the softening and the adhesions are the most prominent.

The theory of the lesions which occur in respect to the nutritive functions, are thus given in the Cerebral Pathology of M. Pinel: "We observe that it is often the muscles of the tongue, larynx, pharynx, œsophagus, diaphragm, the sphincters of the anus and bladder, which first begin to be paralyzed; and it is only afterwards that the inferior extremities, firstly, and then the superior, are affected in their movement. No doubt, for us, but that the affection begins by a lesion of the olivary bodies, or of the nervous fasciculi which come from them; or if it begins at the cerebral periphery, it ought to interest some fasciculus or nervous ramification connected with the olivary body; moreover, that the nerves of the pharynx, larynx, and tongue, originate from the olivary fasciculi, as well as the facial nerve (trembling of the lips and cheeks); that, after Bell, the olivary fasciculi are destined for the respiration, because the pneumogastric, in part, owes to them its origin; that we remark with these patients inertia of the diaphragm, slowness of the respiration, and slowness of the heart; that the lesion of the pneumogastric will explain all these symptoms, as well as the paralysis of the anal and vesical sphincters." We see by this passage that the lesions of the functions of nutrition are multiple; moreover, that the palsy of the internal organs would precede that of the organs of locomotion. I have not found M. Pinel's enunciation confirmed in the works of other authors. If the lesions of the functions of nutrition are not anterior to those of locomotion, it is probable that they begin at the same time. Hypochondriacal ideas, frequent at the beginning of palsy, could, after the theory of M. Moreau (of Tours), be considered as a manifestation of it. The functions of excretion are, moreover, often very irregular; constipation is frequent at the commencement. In some cases, as has remarked M. Baillarger, a sudden retention of the urine arrives to enlighten the diagnosis, till then doubtful. A symptom well known among guardians in asylums is the voracity of the patients, who are about to become paralytic. This symptom is so well known among them, that "to eat like a paralytic," is a common expression in French asylums. This symptom is the more important, as it exists most often from the beginning of the disease. It may be as easily explained by the lesion of the pneumogastric, as the trembling of the facial muscles by the lesion of the facial nerve; but, as has remarked M. Parchappe, it is not necessary to seek for lesions of special nerves, to have an explanation of these facts. It is proved that if the lesions of the cortical part are constant, they do not exist alone, and that those of the white substance most usually accompany them. If we admit, with M. Flourens, that the cerebral lobes are the origin of movement and of sentiment of the whole body, it is easy to comprehend the multiple and simultaneous lesions of the different functions of the human body in general palsy. M. Parchappe, inspired with the works of M.M. Rostau, Lallemand, Bayle, and especially with those of M. Calmeil, writes that when general palsy appears from the beginning with all its characteristic symptoms — special delirium, difficulty in speaking, agitation, lesion of motility — we shall find the specific, anatomical lesion extending over the whole surface of the cortical part of the brain.

If we admit the hypothesis of Gall, adopted by M. Parchappe, *i. e.*, the localization of intelligence and general movement in the cortical surface, then we might comprehend how movement may be affected, and intelligence yet preserved. Nothing, however has yet proved the truth of this assertion; nevertheless, this hypothesis would reconcile itself with observations, if we admitted that one portion of the cortical surface, remaining healthy, could suffice for the preservation of the intellectual faculties; then we might comprehend how the delirium would only appear when the inflammation, even slight, had invaded all the cortical surface; this would also explain the delirium, subsequent or anterior to the palsy.

Nothing proves, in the observations of authors, that the patient has not succumbed from a complication rather than from the general palsy itself. In palsy without delirium, it is probable that if the prolongation of life had permitted the anatomical lesion to completely invade the cortical substance, the true characteristic symptoms, special delirium and palsy, would have been observed. Finally, whether the inflammation remains in the white substance, develops itself around a pathological production, whether it goes from the medulla dorsalis to the encephalon, etc., in all these cases, when the inflammation will have gained a certain extent of the cortical substance, we shall observe symptoms of general palsy ; when the lesion is general, the affection will appear with all its features strongly accentuated.

We will here briefly mention some of the appearances presented at an autopsy. The pia-mater will be found to have lost its transparence, and is now opaque, reddish, from its numerous, tortuous, turgescent vessels. The cellular tissue of this membrane has thickened, and is infiltrated with more or less serous or sero-sanguineous liquid. If we attempt to remove it, it no longer comes away in a normal manner, but in large pieces, or even all at once, owing to its increased solidity, which will be proportionate to the intensity and duration of the disease. While endeavoring to remove the pia-mater from the cortical substance, there will be manifested the great characteristic, anatomical lesion of this affection, *i. e.*, adhesions. These latter are not suddenly formed ; but, as has been well remarked and described by M. Calmeil, are preceded by an inflammatory state, which was most concentrated where the adhesions are the most characteristic. Sometimes these adhesions between the pia-mater and the cortical substance are so intense, that it is impossible to remove the membrane without taking with it more or less of the nervous substance, thus leaving excoriations in the cortical part. These adhesions are principally found on the anterior and inferior faces of the anterior lobes, and around the Fissure of Sylvius. They are less common on the occipital lobes ; when occupying also the cerebellum, they are usually found on the side of its superior and inferior faces. M. Calmeil says : " If the disease has not been intense, the inflammatory state does not appear to have affected the cerebral substance in contact with the meninges to a depth of only a few millimetres ; on the contrary, if the inflammation has passed a certain degree of intensity, then the gray substance of the corpora striata, of the cornua ammonis, and of the thalami optici, is interested in the inflammation. Very often inflammatory foci occupy the circumvolutions which border the fissure of Sylvius, those at the right and left of the falx cerebri, and those corresponding to the inferior part of the anterior cerebral lobes. The same are also seen in the superior, lateral, and convex regions of the middle and posterior lobes of the cerebrum.

The consistence of the cortical substance is softened so much that the slightest touch of the finger or scalpel removes more or less nervous substance. Its coloration is no longer normal, but presents various degrees of redness from congestion; if the disease has been greatly prolonged, the patient not having died during a congestive attack, it may then appear pale or yellowish. The gray substance is reduced by atrophy, and in some cases retains not more than half of its normal thickness.

The white substance of the brain is also congested, having its vessels dilated; the latter remain gaping when a section of the brain is made. If the disease has been of long duration, the white substance may have become hardened and elastic, and by its contractions, have enlarged the lateral ventrics.

The above are some of the appearances visible to the naked eye. If now having made a preparation of the pia-mater, taking a good microscope, we find its vessels considerably enlarged; its capillaries appearing as if covered with fine granulations; its texture presenting extravasated globules of blood, granular cells, and molecules.

The aerosity of the pia-mater under the microscope presents a certain number of free globules of blood, globular cells, ganules, and sometimes pus globules.

M. Calmeil states that in cases of moderate intensity, while the capillaries of the pia-mater are found reddened, greatly developed, and increased in number, yet the texture of this membrane continues to remain with scarcely any infiltration. Also, that the cortical substance is very little, if at all, imbibed with serosity; and, that the agminated cells will only be found on the sides and at the surface of a certain number of vessels. The same author says: "If the malady is intense, we shall find, besides the above intensified, vast arborisations of the vessels of the dura-mater; fibrinous concretions attached to the arachnoid; collections of blood, of serosity, of pus, dispersed in the arachnoidian cavity; sanguineous effusions of the cerebral or cerebellar pia-mater; an excess of congestion, of thickening, of infiltration, of this serous membrane; adhesions, which become established between the pia-mater and the cortical substance; vast excoriations and dispersion of this same nervous substance by an excess of sanguineous injection and a softness of tissue; an induration or softening of the white central substance of the cerebral hemispheres; vast foci of profound, circumscribed encephalitis, situated in some determined region of the cerebellum, of the cerebral hemispheres, or in the auricular protuberance; softening of the central parts of the cerebrum, and sometimes lesions of the spinal cord."

If we contrast the gray substance in a normal condition with the pathological state, there will be seen eight or more vessels dividing and subdividing, thus forming plexuses where one or two vessels in the normal state would be found, thus showing an active process of new formation. Of these vessels, the larger will be seen empty; the others are dilated with globules of blood, rendering the circulation impossible. The calibre of the capillaries is almost always diminished by being exteriorly covered with molecules. These alterations are due to an intense vascularization, and to the extravasation of blastema, in the middle of which the plastic productions finally become organized, thus profoundly modifying the cerebral parenchyma itself. The nervous cells are rare, deformed, disconnected; lose their normal character by losing their contents. The nervous tubes are now lessened and deformed by atrophy, and their contents also escape.

M. Marcé says: "The vascular lesions predominate in the acute cases of general palsy, whilst the plastic effusions, the alteration of the tubes and cells, are more characteristic when the disease has progressed slowly."

Not having time to carefully analyze the histology of the neo-membranes, the cysts, and the granulations of the arachnoid, we can only say that they often exist; that the best theory is to consider the neo-membranes as produced by the secretion from the parietal surface of the arachnoid, and that the different appearances they may present are due to the rupture of some little vessels in them; that the granulations are produced by plastic, albuminous exudations, which are deposited on the free, serous surface as small asperities; that, as says M. Marcé, " The cysts have a variable origin; sometimes they are formed by an effusion of blood into the arachnoidian cavity, determining an inflammation of the serous membrane at the points with which it is in contact; the plastic lymph organizes around the clot, producing its cystic envelope, which after a time may acquire consistence and thickness; at other times, on the contrary, the blood, while effusing, separates a psuedo-membrane situated on the parietal layer of the arachnoid, a new membrane, forming at the superior part of the clot, completes the envelope."

Finally we may remark that the lesion of the cortical substance and the adhesions of the meninges are the capital, characteristic, histological indications of general palsy. Without dwelling longer on the pathological anatomy of this disease, we may assert that always in this malady will be found lesions of the brain and its envelopes, sufficient to satisfy any careful investigator, if he only properly endeavors to find them. It appears to me that the day has passed when the German somatists can truly proclaim, " The majority of mental diseases do not present appreciable alterations." Although our means of investigation are yet so simple as generally not to seize the the primeval cause of lesions so manifest, yet we must not despair; but, on the contrary, advance.

(*To be continued.*)

